# The effects of setarud on the immunological status of HIV-positive patients: Efficacy of a novel multi-herbal drug 

**Published:** 2017

**Authors:** Mehdi Gholamzadeh Baeis, Ghasem Amiri, Mojtaba Miladinia

**Affiliations:** 1 *Young Researchers and Elites Club, Qom Branch, Islamic Azad University, Qom, Iran*; 2 *Infectious disease specialist, Qom University of Medical Sciences, Qom, Iran*; 3 *Nursing Care Research Center in Chronic Diseases, School of Nursing and Midwifery, Ahvaz Jundishapur University of Medical Sciences, Ahvaz, Iran*

**Keywords:** Herbal, Anti-AIDS drugs, IMOD, HAART, HIV, CD4 cell

## Abstract

**Objective:**

This study examines the effect of the addition of IMOD, a novel multi-herbal drug to the highly active anti-retroviral therapy (HAART) regimen, on the immunological status of HIV-positive patients.

**Materials and Methods:**

A randomized two-parallel-group (HAART group versus HAART+IMOD group), pretest-posttest design was used.Sixty patients with indications for treatment with the HAART regimen participated. One week before and 2 days after the treatments, immunological parameters including total lymphocyte count (TLC) and CD4 cell count were assessed.The intervention group received the HAART regimen plus IMOD every day for 3 months. The control group received only the HAART regimen every day for 3 months.

**Results:**

In the intervention group, a significant difference was observed in CD4between before and after drug therapy (CD4 was increased). However, in the control group, the difference in CD4 was not significant before and after drug therapy. The difference in TLC was not significantly different between the two groups before and after therapy. Nevertheless, TLC was higher in the intervention group.

**Conclusion:**

IMOD (as a herbal drug) has been successfully added to the HAART regimen to improve the immunological status of HIV-positive patients.

## Introduction

Human immunodeficiency virus (HIV) damages cellular immune function, reduces the number of CD4 lymphocytes, and finally induces various opportunistic infections (i.e. acquired immune deficiency syndrome (AIDS)) (Dybul et al., 2002 ▶; May and Ingle, 2011 ▶). So far, HIV has claimed the lives of 25 million people in the world, and more than forty million people are currently affected. The virus arrived in Iran in 1986, 23 thousand people have been infected so far, and about 1700 people have died of HIV (Vogel et al., 2010 ▶). 

There are three strategies to deal with HIV, including vaccine administration, antiviral therapy, and strengthening the immune system. Despite many efforts made to produce vaccines, little success has been achieved (Sax and Baden, 2009 ▶). Highly active anti-retroviral therapy (HAART) is the main and standard drug therapy for HIV-positive patients. This regimen affects virus activity and causes CD4 count to increase (Li et al., 1998 ▶), which ultimately leads to the patient’s improved immunity and reduces complications in HIV-positive patients (Lederman, 2001 ▶). At present, the HAART regimen includes more than 30 different drugs of 6 separate classes, such as non-nucleoside reverse transcriptase inhibitors (NNRTIs), nucleoside reverse transcriptase inhibitors (NRTIs), fusion inhibitors, entry inhibitors, protease inhibitors (PIs), and HIV integrate inhibitors (Hammer et al., 2008 ▶). These medicines inhibit the reproliferation of the virus, reduce drug resistance, and eventually revive the immune system (Ho, 1995 ▶). Although the HAART regimen includes a wide range of drugs, each drug combination has certain defects besides its advantages (Cerqueira et al., 2004 ▶; Fellay et al., 2001 ▶; Murphy et al., 2007 ▶; Schwartländer et al., 2001 ▶). 

Treatment based on immune factors such as cytokines, hormones, and interleukins to strengthen the immune system response is a strategy which has been widely studied in the past decade to deal with HIV (Orrell, 2005 ▶). The drugs include Interleukin-2 (Malta et al., 2008 ▶), Remune (Nachega et al., 2011 ▶), IR103 (Nachega et al., 2010 ▶) Pehrg214 (Montessori et al., 2004 ▶), Ampligen (Burgoyne and Tan, 2008 ▶), Avr118 (Barbaro and Barbarini, 2011 ▶), and Revivo (Orsi and d'Almeida, 2010 ▶). Unfortunately, none of the agents strengthening the immune system has led to a significant rise in CD4 count in HIV-infected people. In recent years, Iranian scientists have produced a multi-herbal drug known as the Immune-Modulator Drug (IMOD) to strengthen the immune system. IMOD is an herbal drug which contains *Rosa canina*, *Urtica dioica* (nettle) and *Tanacetum vulgare* (tansy), in addition to flavonoids, selenium and carotenes (Paydary et al., 2012 ▶). IMOD’s mechanism of action is mainly repressive effects on the inflammatory cytokines, but its exact immunological role on the immune cells has not been determined yet.

IMOD decreases TNF-α and can be effective on interleukin-2 and interferon. Moreover, we know that IMOD can have regulatory effects on inflammatory cytokines and the function of B cells, nevertheless, there is not enough evidence on how IMOD affects the activity of T Cells. It should be also noted that the general issue of stimulating the immune system (immunostimulation) by herbs is still unproven (Gertschet al., 2011 ▶). After a period of 90 days of administration, the effects of IMOD on the enhanced immune system remain even 2 years after stopping the drug (Paydaryet al., 2012 ▶). While an immune system booster known as Viosid results in only 11% increase in the lymphocytes, IMOD increases the lymphocytes count by 50%. IMOD can increase CD4 count from 350-400 cell/mm^3^ to more than twice; thus, the patients become resistant to secondary infections which are a leading cause of death in HIV-positive patients (Zabihollahi et al., 2012 ▶). Moreover, the safety of IMOD for HIV-positive patients was shown by Mohraz, Khairandish and their colleagues in Iran (Khairandish et al., 2009 ▶; Mohraz et al., 2013 ▶).

Studies on the effect of IMOD on the immunological status of patients with AIDS are very limited in the literature. In a study by Mohraz (2009) ▶ conducted in 3 phases, AIDS patients were divided into two groups receiving IMOD and HAART, and their CD4 levels were measured (Mohraz et al., 2009 ▶). In another study by Mohraz et al. (2013) ▶, in the fourth phase, the information about 600 patients who had been treated with IMOD was evaluated, and their CD4 and TLC counts were compared in a single-group, pretest-posttest study (Mohrazet al., 2013 ▶).

In this study, we investigated the effect of adding IMOD to the HAART regimen on the immunological status (CD4 and TLC indexes) of HIV-positive patients.

All the available treatments for HIV infection are associated with serious side effects (Siegfried et al., 2010 ▶). Although the HAART regimen reduces mortality in patients with AIDS, it is associated with side effects, toxicity, mental health problems; therefore, it is essential to find new drugs with higher efficacy and fewer side-effects. Approximately 50 to 75% of AIDS patients experience complications caused by HAART (Roca et al., 1999 ▶; Seyed Alinaghi et al., 2012 ▶). Therefore, the use of new herbal drugs with fewer side effects and greater tolerability is essential for patients with AIDS. Today, immune-based therapies have raised researchers’ interest. IMOD is a safe herbal drug among immune-based therapies.

## Materials and Methods

**Figure 1:**
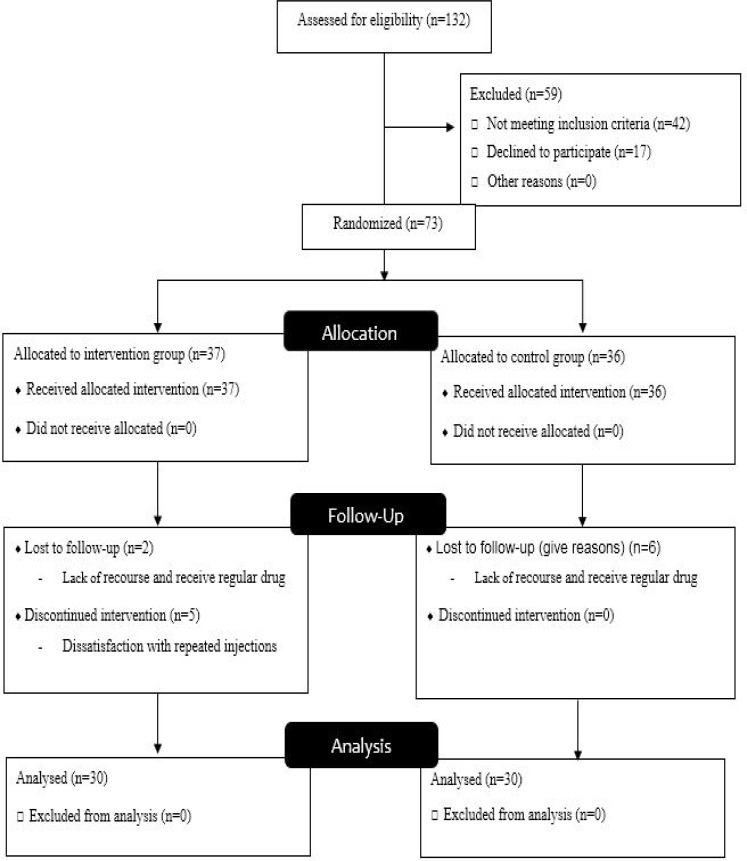
Consort flow chart of randomized controlled trial

A randomized two-parallel-group (HAART versus HAART+IMOD), single-blind (those who examined blood samples), pretest-posttest design was used in this study. Of 132 HIV-positive patients who had medical records at the outpatient Counseling Center for Behavioral Diseases in Qom, I.R. Iran, in 2014, 90 patients met the inclusion criteria and were invited using convenient sampling. Consents were obtained from 73 patients who were enrolled in the study (Figure 1). Participants were randomized into intervention (patients who underwent IMOD infusion in addition to HAART) and control groups (those who received HAART only) using the permuted block randomization method (37 patients in the intervention group and 36 patients in the control group). Finally, after drop-outs were considered, the data for 60 participants were analyzed. The process of investigation was performed over a period of 6 months (August - February 2014).

The inclusion criteria were: 1) two positive ELISA tests and one positive western blot test; 2) patients in the age range of 18–70 years; 3) at least one of the following indications for treatment with the HAART regimen: AIDS-defining diseases, patients with CD4 counts of less than 350, HIV-positive pregnant women, HIV-positive patients in clinical stages 3 and 4 according to classification by the World Health Organization (WHO), HIV-associated nephropathy, and hepatitis B virus infection if it required treatment. For the following cases, we began treatment for patients with CD4 counts of above 350: HIV-RNA of above 100000 copies/mL, CD4 reduction of more than 100 cells/µL/yr, a high risk of cardiovascular disease, and co-infection with hepatitis C virus) (Spencer, 2000). Participants were excluded during the intervention if: 1) they did not attend to receive medication regularly (for 3 sessions), 2) considerable side-effects were observed, 3) the patients were unwilling to continue the study, and 4) their condition deteriorated.

The HAART regimen used in both groups included 1 NNRTI + 2 NRTI. Moreover, to ensure the regular use of the HAART regimen by the patients in both groups, the drug of the HAART regimen was administered to the patients by the researchers on a daily basis (each patient received HAART for 90 days on a daily basis).

IMOD in the intervention group was given as an intravenous injection of an IMOD vial. This vial (4mL=125 mg) was diluted in 100 cc of dextrose water (D/W5%) serum and infused for 30 min every day for 3 months (each patient received IMOD for 90 days on a daily basis) (Mohraz et al., 2013 ▶). The intervention group received IMOD 30 min after receiving the HAART regimen.

One week before and 2 days after the treatment, TLC and CD4 were measured for all participants. Venous blood samples were obtained from right antecubital vein after fasting for 10-12 hr. The number of CD4 cells was measured by fluorescence-activated flow cytometry and TLC was calculated from the complete blood cell count. 

This study was approved by the Ethics Committee of Qom University of Medical Sciences, I.R. Iran. All procedures were in accordance with the ethical considerations of the Declaration of Helsinki. The objectives, the process of investigation, and probable adverse effects of IMOD were explained to all the participants and written informed consent was obtained. Participants were free to exit the study whenever they were willing to. Moreover, the participants were informed that all costs resulting from possible side effects would be supported by the sponsor, and their transportation costs were also paid.

**Statistical analysis**

The data were analyzed using SPSS 17 for Windows (Chicago, IL, USA). In this study, a significance level of 0.05 was considered. Descriptive statistics were used for data analysis (frequencies and percentages for gender, and mean and standard deviation for the continuous variables of age, CD4 and TLC). 

The Shapiro-Wilk Test was used to investigate the normality of results. According to the results of Shapiro-Wilk Test, CD4 results were normal, while TLC results were not. Therefore, independent samples t-test was used to compare CD4 means between the two groups in baseline, and Mann–Whitney U test was used to compare TLC means between the two groups in baseline. In addition, paired-samples t-test and Wilcoxon test were used to compare CD4 and TLC means of pre- and post-tests in each group, respectively.

## Results

In this study, the mean age of participants was 37 ± 9.3 years (intervention: 38.13 ± 8.4, control: 37.06 ± 10.33) and no significant difference was observed between the two groups in terms of age (p=0.663). Furthermore, most of the participants were male (86.6%) and the proportion of genders were equal in both group (24 men and 6 women in the each group). Therefore, the participants were identical in terms of age and gender in the two treatment groups.

The results showed that there was no significant difference between the two group in terms of CD4 mean in baseline (p=0.06). In the intervention group, the mean differences of CD4 before and after drug therapy showed a significant difference (mean difference=172.3, p=0.001). However, in the control group, the mean differences of CD4 before and after drug therapy were not significantly different (mean difference=4.3, p=0.87) (Table 1). 

**Table 1:** Comparison of CD4 mean [cell/mm^3^] between pretest with post-test treatments

**Groups**	**Time**	**Mean ± SD**	**p**
**HAART+IMOD**	Before	292.7±218.2	0.001*
	After	465.0±235.6	
**HAART**	Before	401.8±221.9	0.87
	After	406.1±195.9	

- SD (Standard deviation)

- Paired t test was used.

* statistically significant (p<0.05)

The results of CD4 cut-off in the intervention group showed that the mean difference of CD4 was significant before and after the intervention both in patients with CD4 counts of below 200 (p=0.017) and patients with CD4 counts of over 200 (p=0.005). However, the HAART+IMOD regimen had a greater impact on patients with higher CD4 counts (MD: 175.7 vs. 351.6) (Table 2).

**Table 2:** Comparison of CD4 mean [cell/mm^3^] between pretest with post-test treatments in patients with CD4 count less than 200 cells/mm3 and patients with CD4 count above 200 cells/mm3 in the intervention group

**CD4 count**	**Time**	**Mean ± SD**	p
**patients having CD4 count less than 200**	Before	116.5±44.9	0.017*
	After	292.2±181.2	
**patients having CD4 count above than 200**	Before	213.7±49.0	0.005*
	After	565.3±205.8	

- SD (Standard deviation)

- Paired t test was used.

* statistically significant (p<0.05)

According to the results, there was no significant difference between the two groups in terms of TLC mean in baseline (p=0.403). Based on Wilcoxon test, there was no significant difference in the mean differences of TLC before and after drug therapy in the two groups (intervention group‘s mean difference=293.6 and p=0.06, and control group’s mean difference=70.5 and p=0.436). However, the mean difference of TLC was higher in the intervention group compared to the control group (Table 3).

**Table 3:** Comparison of TLC mean [cells/μl] between pretest with post-test treatments in the each group

**Groups**	**Time**	**Mean ± SD**	p
**HAART+IMOD**	Before	1655.4±742.0	0.06
	After	1949.0±673.1	
**HAART**	Before	1833.3±886.1	0.436
	After	1903.8±671.2	

- SD (Standard deviation)

- Paired t test was used.

* statistically significant (p<0.05)

## Discussion

Results showed that adding IMOD to the HAART regimen can further improve the immunological status of HIV-positive patients (as it increased CD4 and TLC counts). One of the indicators evaluated to investigate the immunological status of patients was the CD4 count. Several studies have shown that CD4 count is the strongest predictor of disease progression and an estimator of patient’s survival. First, CD4 count is an independent and reliable criterion to assess the treatment outcome, it is the most important factor for decisions about starting antiretroviral therapy. Second, CD4 count is a relatively simple and objective sign to continue the patients’ treatment (Egger et al., 2002 ▶; Mellors et al., 1997 ▶). Third, typically, CD4 increases rapidly in the first months of treatment (Gengis and Deeks, 2009 ▶). Finally, CD4 count is especially cost-effective in developing countries (Lutwama et al., 2008 ▶; MacLennan et al., 2007 ▶). Hence, CD4 lymphocyte count is the best tool to measure the immune performance of the host cell (Spencer, 2000 ▶). Thus, in the present study, according to the available facilities and funds, we measured the CD4 count of patients. 

Results showed that CD4 levels increased significantly after 90 days (p=0.001) in the intervention group who received IMOD in addition to the HAART regimen. In a study conducted in 3 phases by Mohraz et al. (2009) ▶ in Iran, results showed that an increase in CD4 mean was 2-3 times greater in the intervention group (who received IMOD) than the control group (who received HAART). In this study, a slight increase was observed in CD4 in the control group who received the HAART regimen only; however, the mean difference was not significant (p=0.87). The difference was significant in the control group in the study by Mohraz; however, the mean difference was greater in the intervention group than in the control group. Therefore, similar to our study, the effectiveness of IMOD was greater than the HAART regimen. The difference between our study and the one done by Mohraz was that our intervention group received HAART+IMOD, while the intervention group in Mohraz’s study received IMOD only (Mohraz et al., 2009 ▶). In another study by Mohraz et al. (2013) ▶ on 600 patients, results showed that the daily intake of IMOD significantly increased CD4. It was a single-group study, and IMOD was not compared with HAART (Mohraz et al., 2013 ▶).

In the present study, the mean difference was statistically significant in the intervention group before and after the intervention both in patients with CD4 counts of below 200 (p=0.017) and patients with CD4 counts of over 200 (p=0.005). However, the HAART+IMOD regimen had a greater effect on patients with higher CD4 counts. Similar to our study, in the study by Mohraz et al. (2013) ▶, the mean difference was significant both in patients with CD4 counts of below 200 and patients with CD4 counts of between 200 and 400, before and after the intervention (Mohraz et al., 2013 ▶). Nevertheless, in the study done by Mohraz, unlike our study, IMOD regimen had a greater effect on patients with lower CD4 counts. This difference is likely due to the differences in sample size and the inclusion criteria. Hence, further research is needed in this area to determine on what CD4 count range, IMOD has the greatest efficacy.

TLC was the other indicator that we evaluated. According to the literature, TLC can be considered as a suitable pre-indicator of CD4 count, and there is an association between TLC and CD4 which can be used specifically in deprived conditions (such as patients with low income) (Mwamburi et al., 2005 ▶; Schreibman and Friedland, 2004 ▶). It can also be used as an alternative potential marker for immune system performance (Schreibman and Friedland, 2004 ▶). Therefore, TLC was evaluated along with CD4. Although, in this study, the mean difference of TLC before and after intervention was not significant in either group, in the study done by Mohraz et al. (2013) ▶ in which the patients received IMOD, the daily intake of IMOD significantly increased TLC levels (Mohraz et al., 2013 ▶). Nevertheless, it is noteworthy that the mean difference of TLC was higher in the intervention group (receiving IMOD) than the control group.

HIV-positive patients who had indications for HAART therapy are exposed to numerous risks such as complications of antiretroviral drugs (Fellay et al., 2001 ▶; Paydary et al., 2012 ▶), viral resistance, failure of treatment (Bangsberg et al., 2004 ▶; Khorram Khorshid et al., 2008 ▶; Lucas, 2005 ▶) , and high costs (Ho, 1995 ▶). IMOD increases CD4 count even 6 months after the discontinuation of the drug (Mohraz et al., 2009 ▶) with few complications (Azonov et al., 2008 ▶; Khairandish et al., 2009 ▶; Khorram Khorshid et al., 2008 ▶; Khorshid et al., 2008 ▶) and high effectiveness (Orsi and d'Almeida, 2010 ▶). It is recommended that IMOD should be considered as a part of treatment in developed and developing countries. Since the oral form of IMOD is still not used and patients avoid multiple daily injections, further research is recommended on production of the oral preparation of IMOD. 

This study had a number of limitations:

 1) It was limited to a specific geographic area, reducing the generalizability of the results.

 2) Long-term effects of IMOD were not examined because a large number of patients quit. Therefore, further research is recommended to be conducted in other geographical areas to investigate the long-term effects of IMOD

3) Many factors such as comorbidities, prolonged psychological stress, and the use of concomitant medications such as interferon were not controllable by the researchers and may have affected the parameters investigated in this study

4) Another limitation of this study was that the exact experimental mechanism and the effect of IMOD on the immunological status of HIV-positive patients were not specified, because the use of this drug in the HIV-positive patients is still in the primary phases. So, we suggest future studies to be conducted on the exact mechanism and effect of IMOD on the immunological status of HIV-positive patients.

Given the increasing prevalence of AIDS worldwide, it is essential to improve the conditions of AIDS patients and promote their quality of life. Thus, it is essential that these patients use new drugs with higher efficacy and fewer side-effects. 

In this investigation, IMOD has been successfully added to the HAART regimen to improve immunological status of HIV-positive patients. According to the results, the use of IMOD in HIV-positive patients, IMOD as a herbal drug is a safe and effective treatment and can be included in the therapeutic regimen of HIV-positive patients.

**Implication for practice**

Immunologists can use IMOD along with the HAART regimen to improve the immunological status and control the complications in AIDS patients. It can be considered as a good alternative to complicated treatments. This herbal drug can also increase the tolerability of treatment regimens in these patients. Future studies should investigate the effect of IMOD on other immunologic parameters such as CD8. IMOD treatment period should also be studied to examine its long-term effects. In addition, we suggest that IMOD could also be considered as an individual medication without the HAART regimen.
